# The Normative Role of the Circular Plastics Alliance in the EU’s Transition towards a European Circular Economy for Plastics

**DOI:** 10.1007/s43615-024-00380-8

**Published:** 2024-05-27

**Authors:** Amy O’Halloran

**Affiliations:** School of Law, University College Cork, Cork, Ireland

**Keywords:** Circular Plastics Alliance, Standardisation, Law, Regulation, European Union, Plastic Recycling, Legal Theory

## Abstract

The global pollution and waste crisis presents us with environmental and economic challenges which if not properly addressed could destabilise or threaten the survival and welfare of societies. The European Union is responding to the waste and pollution crisis through its circular economy agenda that adopts a broad life-cycle approach to the regulation of plastics from production, consumption, disposal, and recycling. To operationalise its agenda, the European Union seeks to *inter alia* mobilise all actors towards the objective of improving the economics of plastic recycling. Given the potential for conflicts and disputes to proliferate across a broad range of societal actors and interests, it is perhaps not surprising that when we examine the evolving EU legal and normative framework for a circular plastics economy, we observe a polycentric governance arrangement that includes the EU institutions, the Circular Plastics Alliance (CPA), and European standardisation organisations (i.e. CEN and CENELEC). The normative interactions amongst these governance bodies will not easily be unveiled and understood if we enclose our perspectives and analyses within the limits of traditional legal paradigms that *only* focus upon the formal law-making processes that flow through the European Parliament, Council, and Commission. However, by applying Karl Llewellyn’s law-jobs theory in this article, it is possible to analyse how a multiplicity of governance bodies perform certain *legal functions* that are contributing to the development of regulatory order for a European circular plastics economy. This article sets out a number of key findings in relation to the evolving legal and normative framework for a European circular plastics economy pertaining to the role of the CPA in framing problems, theorising solutions, and shaping the pathway of normative development towards a European circular plastics economy. To date, the CPA has identified obstacles to the expansion of the European recycled plastics market, and mapped the areas in need of standardisation if such obstacles are to be overcome This work by the CPA has prompted the European Commission to submit a standardisation request to the CEN and CENELEC calling for the development of harmonised standards to facilitate greater plastic recycling. While compliance with CEN and CENELEC standards would be voluntary, such standards could interact with the EU’s proposed *Ecodesign Regulation* and any delegated acts adopted thereto, thereby creating legal obligations for a wide range of actors across plastic value chains.

## Introduction

Certain arenas of human life such as technological development may at first appear to be entirely technical in nature wherein, they possess no distinctly legal element. However, the American jurist Karl Llewellyn asserted that “there is almost no part of culture which is not also ‘legal’ in nature (whatever else it may be as well)” [[1] p 1377]. Adopting a functionalist legal perspective, Llewellyn argued that because social interactions are attended by risks of conflict or grievance (i.e. ‘trouble’), the law is always in the background regardless of how seemingly non-legal the context [1]. He reasoned that when trouble arises the law usually emerges to perform important functions such as resolving disputes, channelling conduct, rechannelling conduct, reorientating societies towards collective objectives (i.e. ‘net positive drive’), and restoring and maintaining social harmony. As a result, Llewellyn concluded that law tends to be “mixed into any coordinated action” such that the law “infests” human culture [[1] p 1377].

The great environmental crises of our time (i.e. climate; biodiversity loss; pollution and waste) [2, 3] have engendered ‘trouble’ to a critical level today. These crises, and the risks they pose to *all* societies are provoking coordinated action and normative development in order to avert, mitigate, or adapt to these unfolding crises. Such normative development is sometimes occurring through new organisational forms that don’t always sit easily within the legal and political paradigms of State and international law [4]. For example, in the context of the plastic pollution and waste crisis, the European Union (EU) seeks to implement a circular economy agenda partly by utilising industrial alliances as pioneers in normative development [5]. Industrial alliances have been described by Huang et al. as an organisational form within industry that is usually purposed towards conquering technological objectives through the mobilisation of industrial actors [6]. In relation to the EU’s circular economy ambitions, the establishment of industrial alliances across a number of key sectors is intended to respond to some of the technological, operational, and economic challenges associated with realising a European circular economy [7]. In the plastics sector, the Circular Plastics Alliance (CPA) was established in 2018 [8], for the purpose of fostering the growth of the European recycled plastic market through *inter alia*, progressing normative development towards design-for-recycling standards and guidelines for plastic products, components, and packaging [5].

Novel organisational mechanisms such as industrial alliances and the complex entanglement of social and environmental crises to which they respond, add to existing exigencies that impel scholars (including legal scholars) to “rethink basic concepts in the organisation of our societies” [[9] p 25]. As industrial alliances are not formal law-making forums like State legislatures or the EU institutions, legal scholars are faced with a more polycentric governance context within which they must move beyond “positing simple systems to using more complex frameworks, theories, and models to understand the diversity of puzzles and problems facing humans interacting in contemporary societies.” [10] p 641]. Accordingly, this article will assume the task of confronting the conceptual and methodological challenges of understanding the role of the CPA within the evolving legal and normative framework for a European circular plastics economy by adopting the functionalist legal perspective of Karl Llewellyn’s ‘law-jobs theory’ [1]. Llewellyn’s theory was developed in the 1930’s to address the conceptual and methodological challenges of analysing the legal and normative organisation of indigenous societies in North America whose institutional structures and practices differed from the types of legal and political systems that were typically found in Western States [11]. The law-jobs theory, for which William Twining articulates a contemporary interpretation [11], provides an illuminating research paradigm for examining functionalist aspects of law and law-like phenomena that do not fit neatly within the formal unitary law-making structures of particular States [11]. The law-jobs theory also facilitates analyses of combinations of law and law-like phenomena as may occur in a polycentric governance arrangement such as the EU wherein the EU institutions, European standardisation organisations, and the CPA mutually interact in the development of rules and standards for a European circular economy for plastics [12]. Methodologically, the law-jobs theory may be described as a functionalist research approach that seeks to identify *the point* of particular institutions, institutional practices, or institutional configurations from a legal perspective [11]. In this way, the law-jobs theory is concerned with the provision of legal order within society and the functions that must be performed to achieve such order (e.g. channelling of conduct, dispute resolution). It should be emphasised that the functionalist approaches of Llewellyn and Twining are based upon an understanding that law has a distinctive role in social organisation, wherein law is *primarily* orientated towards the provision of order rather than the various instrumental objectives that are pursued through the institution of law. Indeed, there are many different objectives that may be pursued in furtherance of establishing a circular plastics economy, and there is no universal conception of what a circular plastics economy entails. Scholars have shown that articulations of a circular economy are often political statements rather than value neutral statements [13, 14]. Moreover, Yalcin et al. have observed that the plurality of ideas around circularity mean that particular conceptions of a circular economy may range from material-focused, to plastics-economy focused, to socio-ecological systems-focused. Material focused conceptions may see recycling as a priority towards achieving resource-efficiency, climate-neutrality, and competitiveness [15]. Such conceptions are closely aligned to the EU’s European Plastic Strategy [16] which seeks to mobilise industry towards more circular patterns of production. Conceptions that focus on the plastics-economy seeks to emphasise the benefits of establishing circularity at scale [15]. These conceptions may assert the importance of plastics utilisation in contemporary life and argue that significant economic benefits (e.g. new jobs, greater revenue, reducing dependencies) may be accrued from innovation and economic growth that is orientated towards circularity [15]. Such a conception of the circular plastics economy sees business and industry as key actors in an economy-wide transformation towards circularity, and there are aspects of this approach evident in the EU’s desire to mobilise industry and society towards circular economics. Socio-ecological systems-focused conceptions of a circular plastics economy may see plastics as unnatural materials that cause significant environmental harm and that paradigmatic change is needed across society to achieve sustainability. Unlike the plastics-economy conception, the socio-ecological systems-focused conception of circularity is driven by environmental concerns as opposed to economic opportunity [15]. Those who subscribe to socio-ecological systems focused conceptions of circularity may be sceptical that entropy allows for repetitious recycling of plastic materials, and that economic growth can be decoupled from environmental harm [15]. The viewpoints that follow from this may seek to criticise other conceptions of circularity which they perceive to be overly technocratic, or business orientated, and whose ultimate purpose is considered to be the solidification of existing economic power structures in society [15, 17]. Views informed by socio-ecological systems-focused conceptions of a circular plastic economy are also likely to be sceptical that the EU’s circular economy agenda will adequately ensure environmental protection and sustainability. Accordingly, the typology of circularity discourse developed by Yalcin et al. reveals that different actors may articulate different conceptions of circularity, and advocate for competing strategies, that are aligned with divergent aspirations of a circular plastics economy [15]. Moreover, within these discourses the prevailing urgency for change may contrast; with some discourses on circularity seeking to promote incremental reforms to institutions and practices, whereas other discourses may pursue transformational change by challenging the underlying power and market relationships in the plastics sector and society [13, 14]. These contests between competing conceptions of a circular plastics economy are reflective of the struggles that occur within law-making, particularly where significant interests are at stake.

However, law is an institution that is distinct from the instrumental objectives and policies that prompted the making of law [18]. According to Llewellyn and Twining, law is primarily orientated towards the provision of order [1, 11]. Therefore, law is an institution that may serve a different policies, purposes, interests, and aspirations (e.g. environmental protection, social protection, economic growth, consumer protection) at different times [13, 14]. However, regardless of the instrumental objectives that prompted the making of law, the role of law within societies is the provision of order [1, 11]. Indeed, there may be other ways and means of achieving particular instrumental objectives that do not involve law (e.g. economic activities, education, political activism). However, when such objectives are institutionalised through law, then the *point* is the provision of order. Through its focus on the functions that contribute to the provision of order, Llewellyn’s law-jobs theory provides a heuristic framework for examining the functional role of the CPA within what is becoming an increasingly complex EU regulatory order. The law jobs theory facilitates an analysis of the functions performed by the CPA that contribute to the provision of order within the EU regulatory order, while also avoiding any conflation of the CPA’s role with the particular instrumental objectives pursued by the CPA (e.g. identifying and addressing the obstacles to the expansion of the European recycled plastics market).

The law-jobs theory is based upon the premise that all citizens and enterprises are members of societies and social groups [1]. From this premise the theory postulates that there are six functions typically performed by law that are necessary for the continuing survival and welfare of social groups, namely (i) dispute resolution, (ii) the channelling of conduct and expectations, (iii) the rechannelling of conduct and expectations, (iv) the allocation of authority to officials and institutions, (v) the provision of ‘net positive drive’ (i.e. establishing collective aspirations and policy objectives for a social group), (vi) and the juristic method for decision-making [1]. According to Llewellyn and Twining, the performance of each of these law-jobs contributes to the provision of order and maintaining harmony within societies. The most relevant of these law jobs or functions for pursuing an analysis of the EU’s engagement with the CPA is firstly, the provision of net positive drive by the EU institutions through their circular economy agenda that steers the output of industrial alliances towards circularity in the European plastics sector. Secondly, the rechannelling of conduct and expectations is a law-job performed partly by industrial alliances such as the CPA, which is tasked with identifying obstacles to the realisation of a European circular plastics economy and then, stimulating normative development in the direction of more circular patterns of behaviour. Thirdly, the work of the CPA in progressing normative development in the area of European standardisation could help to facilitate the channelling of conduct and expectations through the interaction of European Standards with proposed EU legislation (if adopted). The effective channelling of conduct and expectations may require legal authority to enforce rules and standards in the event of non-compliance. While compliance with European Standards is voluntary, the interaction of such standards with EU legislation can provide important support to the implementation of EU law, thereby facilitating the channelling of conduct and expectations. It should be noted that while the CPA is orientated towards identifying problems and fostering normative development, the CPA is not an direct participant in the performance of law-jobs such as dispute resolution, allocation of authority, the channelling of conduct and expectation, and the institutionalisation of juristic methods. The CPA does not have the legal or regulatory authority to channel conduct and expectations through the enforcement of rules and standards. The CPA is not an adjudicatory body and consequently the CPA does not undertake dispute resolution. The CPA has not been vested with the legal and political authority to allocate authority to others. Lastly, in contrast to systems of appellate courts, the CPA is not a juristic body which is engaged in the development of legal doctrine and culture. However, despite these functional limitations, the CPA is playing a salient role in the rechannelling of conduct and expectations for the European recycled plastics market. The contribution of the CPA to the performance of this function also helps to *facilitate* the performance of law-jobs by other bodies within the EU regulatory order such as the EU institutions and European standardisation organisations. For example, the rechannelling of conduct and expectations through the development of European Standards is an enabling condition that helps to facilitate the channelling of conduct and expectations through EU law, which will prevail over individual interests in the event of a conflict or a dispute [1]. Accordingly, this article will firstly analyse the provision of net positive drive by the EU institutions, and then examine the rechannelling of conduct and expectations by the CPA, before considering the practical implications of these functions in relation to the channelling of conduct and expectations through an analysis of the normative interactions between the CPA, European standardisation, and EU legislation.

To analyse the role of the CPA within the EU regulatory order, a desktop study was conducted based on institutional documentation pertaining to policies, strategies, procedures as well as EU legislation (and proposed EU legislation). It should be noted that the study did not include primary data from interviews with officials or stakeholders. The following 11 EU institutional documents were considered; the European Commission’s *First Circular Economy Action Plan* [19]; *Second Circular Economy Action Plan* [20]; *European Strategy for Plastics* [16]; *European Green Deal* [21]; the EU’s *Industrial Strategy* [22]; the updated EU *Industrial Strategy* [23]; The ‘Blue Guide’ on the implementation of EU product rules [24]; the European External Action Service (EEAS) *Global Strategy for Foreign and Security Policy* [25]; the Vademecum on European standardisation [26]; the Commission’s 2022 standardisation request, and draft standardisation request, that both called for the development of harmonised standards for the regulation of product design, the quality of sorted plastic waste, and the quality of plastic recyclates [27, 28]. The study also analysed 4 documents produced by the CPA, namely: the Declaration of the Circular Plastics Alliance [29]; Roadmap to 10 Mt recycled content by 2025 [30]; Guidance on Waste Definitions [31]; and the Design for Recycling Work Plan [32]. Given the potential for normative interactions between European Standards and EU legislation, this article also engages with EU legal instruments such as delegated acts adopted by the Commission [33, 34], as well as the *Ecodesign Directive* [35], the proposed *Ecodesign Regulation* [36], and the proposed *Packaging and Packaging Waste Regulation* [37].

The data in the forgoing documents is categorised and analysed in accordance with the framework of Llewellyn’s law-jobs theory. EU policies and strategies are captured under the heading; provision of net positive drive which facilitated an analysis of how these policies and strategies steer society along certain pathways of change. The normative work of the CPA, and the EU’s standardisation request, are captured under the heading; rechannelling of conduct and expectations which identifies and analyses law-making and norm-making processes. EU legislative documents are analysed in relation to their potential interaction with proposed European Standards for plastic recycling. Such analysis provides a vision of how European Standards might support the implementation of new EU legislation that will impose legally-binding obligations that are capable of channelling the conduct and expectations of economic and societal actors. The next section of this article will begin by looking at the existence of, or the risk of ‘trouble cases’, which Llewellyn considered to be a prerequisite for the emergence of law. In other words, Llewellyn saw the institutionalisation of law as a response to problems that could destabilise or threaten social life.

### The Trouble Case – Plastic Waste Generation and Pollution

Llewellyn expressed the view that one of the fundamental functions of law in society is resolving and avoiding conflicts and disputes, which if amplified and multiplied, could potentially threaten the survival or welfare of society. Llewellyn described such conflicts and disputes as trouble cases [1]. If trouble cases remain unresolved and fester, then they have a destabilising effect on social harmony, which could in time potentially lead to the dissolution of social groups [1]. The deterioration of our environment presents many such trouble cases including the crises of climate, biodiversity loss, and pollution and waste [2]. The pollution and waste crisis has been driven in part by the exponential growth in plastic production, and plastic waste generation. Indeed, the proliferation of plastic waste and pollution and the attendant risk to society poses a trouble case that has roused the concern and angst of officials, policy makers, environmentalists, and wider society [38]. For example, commenting on the particularly deleterious effects of plastic pollution on the marine environment, the United Nations Environmental Assembly recently expressed the view that marine plastic pollution is a “serious environmental problem at a global scale.” [[39] p 1] Some scholars have described marine plastic pollution as a “wicked” problem owing to the difficulties and the interdependencies that are associated with addressing the problem [[40] p 282]. Indeed, the 27th edition of the *Oxford Atlas of the World* describes the prevalence of plastic material in the environment as follows;

“Plastics pervade every environment on Earth. Plastic waste can be found in the remotest areas, far from its place of origin.”[ [41] p 9].

The negative environmental impacts of plastic litter and debris can pose risks to wildlife, human health and marine activities. For example, marine animals can become entangled or asphyxiated upon coming into contact with plastic debris. There are also risks associated with plastic debris being ingested by marine animals who mistake it for food [42–45]. Once ingested, plastic materials and toxins can bioaccumulate in marine animals [46–50], and thereafter pose a threat to human health through the food chain [51]. Several species of fish consumed by humans have been found with toxic plastics in their stomachs (e.g. mackerel, striped bass and Pacific oysters) [52, 53]. There is also a growing concern regarding the risks of toxins being transferred to humans directly from plastic products and packaging [42, 54], because chemicals added to plastics with a view to improving product performance (e.g. plasticizers, stabilizers, and flame retardants) can pose risks to human health [42]. Plastic pollution can also interfere with human activities and amenities and there are various examples of plastic pollution impacting upon fishing [55, 56], shipping and transport [55], and tourism [55].

Despite a number of known negative environmental impacts and risks, it should be remembered that there are also significant benefits accrued through the utilisation of plastics in meeting important social needs. Plastics are “inexpensive, lightweight, strong, durable, corrosion resistant, with high thermal and electrical insulation properties.” [[42] p 1973] By virtue of possessing such properties, various plastics have facilitated technological advances that have benefited society in the areas of vehicle manufacture, fuel efficiency, electronics, utilities, medicinal products, and food preservation [42]. Given the utility of plastics, some scholars have expressed the view that “[a]ny future scenario where plastics do not play an increasingly important role in human life therefore seems unrealistic.” [[57] p 1983]. Plastics make our lives “easier, safer, healthier, and more mobile” [[58] p 14]. Indeed, projections indicate that plastic production is going to increase significantly in the coming decades [59, 60].

Given the utility of plastics in contemporary life, it is not surprising that the plastics industry occupies a position of significant economic importance. Indeed, the United Nations Conference on Trade and Development (UNCTAD) has estimated the value of the global plastics sector at over US$1 trillion in 2018. This figure represents about 5% of the value of global trade and these statistics do not account for the value and volume of hidden plastics embedded in products and packaging [61]. In Europe in 2019, the plastics industry (producers and convertors) employed over 1.5 million people, and generated an aggregate sectoral turnover of over €350 billion which created a positive trade balance of €13.1 billion in then EU28. The sector also contributed around €28.6 billion to public finances and welfare [62].

Accordingly, the utilisation of plastics provides important social and economic benefits for contemporary society. However the way that society manages plastic materials is attended by significant environmental and human health risks. As a result, public authorities and private actors face the challenge of facilitating the beneficial utilisation of plastics while also mitigating or eliminating the risk of harm to the environment and human health. This challenge is made more difficult given that the current scale of waste generation is unsustainable. The Organisation for Economic Co-operation and Development (OECD) claims that 22% of plastic waste is mismanaged [63]. Indeed, the pressures on the world’s waste management systems became particularly salient in recent years following the introduction of waste import restrictions by China (i.e. ‘Green Sword’ policy) [64, 65], Malaysia [66, 67], and Turkey [68]. Such restrictions on waste imports have in part contributed to a 50% reduction in waste exports leaving the EU27 and United Kingdom between 2016 and 2020 [58]. In addition to the environmental risks, the management of European plastic waste presents an economic challenge in relation to the efficient management of resources. The generation of plastic waste and the loss of plastic materials in Europe remains high with around 31% of plastic waste going to landfill, and around 39% going to incineration in 2016 [16, 69]. The European Commission has estimated that around 95% of the material value of plastic packaging material is lost after a single-use, representing an estimated financial loss of between US$80–100 million annually [16, 69]. The Commission has also estimated that the adoption of circular economic practices that keep plastics in the economy for longer would result in an increase of 0.5% in EU Gross Domestic Product (GDP) by 2030, and would lead to the creation of around 700 thousand new jobs, while also providing significant savings for manufacturers [70].

The foregoing synthesis of some of the main advantages and disadvantages of the utilisation of plastics in contemporary societies illustrates that while plastics contribute positively to human welfare and the wider economy, plastics may also have a destabilising effect on social groups if there is a failure to address the significant environmental and economic risks associated with plastic usage. Accordingly, society’s utilisation of plastics poses a classic Llewellyn trouble case that possesses the potential to generate many conflicts and disputes between competing interests as they face difficult environmental and economic challenges.

## How the Law Can Address Trouble Cases

Karl Llewellyn explained that social life requires that individual behaviour and expectations are channelled in order to avoid the proliferation of conflicts and disputes that could imperil the welfare and survival of social groups [1]. Law plays a key role in the channelling of conduct within society as legal rules and standards are enforceable against recalcitrant persons in the event of conflicts or disputes. The enforceability of legal rules and standards means that they have *teeth* which are capable of *biting back* against those persons who seek to thwart the *legal* expectations of society [1, 71, 72] However, in order to channel the conduct of individuals towards society’s expectations, it is firstly necessary to determine the lines along which behaviour and expectations are to be channelled [1]. In a State legal system, this function of rechannelling conduct and expectations is typically performed by those bodies or actors that are vested with law-making capacity, such as legislatures, judges, officials, and regulators etc [1]. .

However, law-making in relation to the current plastic pollution and waste crisis is intensely complex. Law-makers are faced with an intricate governance context that: encompasses many sectors (e.g. agriculture, construction, packaging, electrical and electronic equipment); traverses all phases of the plastic life-cycle (e.g. extrusion, conversion, production, consumption, product usage, end-of-life) [73, 74]; and pertains to all stages of the waste management process (e.g. disposal, collection, sorting, recycling, landfill) [74]. Accordingly, the plastic pollution and waste crisis challenges law-makers to rechannel conduct and expectations across many different parts of society. As will be explained below, the EU seeks to address this governance challenge partly through the strategic use of the CPA as a forum to progress normative development across the full value chain for plastics, thereby encompassing relevant aspects of plastic production, branding, retail, as well as waste collection, sorting, recycling etc [30]. 

**Fig. 1:**
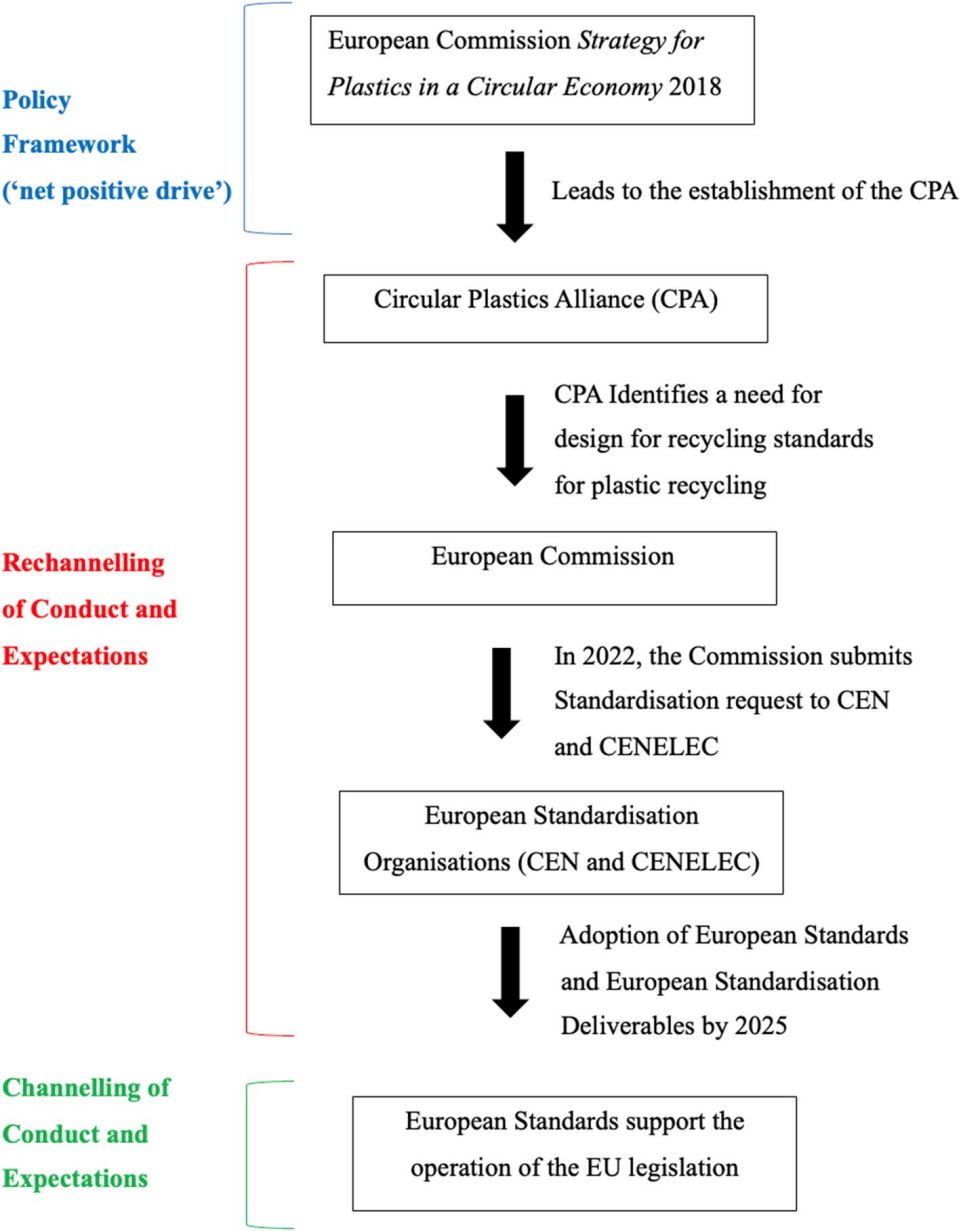
Illustrates the establishment of the CPA and the thrust of its influence in the rechannelling of conduct and expectations towards the development of European Standards by CEN and CENELEC. Such standards, if adopted, may support the implementation of EU legislation which channels the conduct and expectations of actors across plastic life-cycles

If the conduct and expectations of a broad spectrum of European society are to be rechannelled towards circularity, then there must firstly be a clear idea of what a ‘circular economy’ means or symbolises in a European context. According to Llewellyn, the coalescence of society around collective ideals and objectives is a prerequisite to *orientating the direction* of normative development within a social group [1]. He explained that to effectively coalesce society in this way requires the provision of a net positive drive that is capable of mobilising and steering society in the direction of collective ideals and objectives. A net positive drive describes the collective impetus or incentive that is necessary to propel normative development and policy towards certain collective objectives minus the centrifugal tendencies of individuals or sub-groups which might pull in the direction of their own particular interests [1]. Accordingly, the net positive drive of a social group conveys the collective aspirations of that social group, and the group’s institutional efficiency in striving towards such aspirations [1, 12]. As a result, the provision of a net positive drive is a function typically performed by political and legal institutions that claim to speak for the whole of society (e.g. governments, legislatures, judges) [1]. In terms of establishing a European circular economy, net positive drive is provided by the EU institutions through their circular economy agenda that is intended to coalesce European society around the long-term overarching objective of establishing a European circular economy for plastics [1]. Figure 1 below illustrates the functional role of the CPA and the EU institutions within the EU governance configuration for a circular plastics economy.

## Provision of Net Positive Drive by the EU Institutions

The establishment of a European circular economy will require that the actions of many different actors are coordinated and synergised [16, 21]. The EU institutions are well placed to provide the net positive drive capable of orchestrating collective action because institutions such as the Commission are firstly, in a position to articulate a common ideal of a *European* circular economy, and secondly, the Commission has the ability to pursue policy initiatives and strategies that mobilise actors towards the realisation of a European circular economy.

While there are many different articulations of what the concept of ‘circular economy’ represents [13–15, 75, 76], it is the EU institutions that determine what a circular economy means for the European Union as a whole. For example, the Commission’s *First Circular Economy Action Plan* adopted in 2015, articulated the EU’s aspiration of establishing a European circular economy “where the value of products, materials and resources is maintained in the economy for as long as possible, and the generation of waste minimised” [19] p 1]. This vision of a circular economy encompasses the entire product life-cycle from the design of a product to its end-of-life phase [19]. The Commission’s *Second Circular Economy Action Plan* which was adopted in 2020, explains that for European citizens, a circular economy aims to provide “high-quality, functional and safe products, which are efficient and affordable, last longer and are designed for reuse, repair, and high-quality recycling.” [20] Sect. 1]. Accordingly, the Commission’s current vision of a European circular economy could be said to entail transformative changes to market practices that generate circular patterns of production, consumption, and disposal by means of: reducing the consumption of resources; promoting the use of recycled materials; and facilitating the reuse and repair of products [76]. Such a vision could be said to provide a general compass which orientates European society towards circularity.

However, the provision of net positive drive involves more than just the articulation of collective ideals and aspirations. A net positive drive also requires that actors are mobilised towards the realisation of collective objectives. For example, the Commission’s *Second Circular Economy Action Plan* seeks to guide the legislative agenda of the EU by streamlining regulation and establishing a sustainable product policy framework [20]. In accordance with that action plan, a proposal for a new *Packaging and Packaging Waste Regulation* has been advanced by the Commission and the Parliament. If adopted, the proposed Regulation would require that actors along plastic value chains ensure that all packaging is recyclable by the year 2030, and recyclable at scale by 2035 [37, 77]. The *Second Circular Economy Action Plan* also seeks to accelerate the implementation of the *European Green Deal* [21] which is an economic strategy that is intended to deliver a more sustainable future for European citizens by *inter alia* fostering greater resource-efficiency, improving competitiveness, and ultimately decoupling economic growth from resource use [19, 21, 78]. The Commission has acknowledged that the transformation of the European economy in this way will need to draw upon the full collective capacity of the EU, including the mobilisation of industry [21]. As a result, the Commission has sought to pursue a greater engagement with industry [21], through its new *Industrial Strategy* which was published in 2020 [22], and updated in 2021 [23]. This strategy seeks to mobilise industry towards circularity by formalising cooperation through industrial alliances. So far, the Commission has established a number of industrial alliances across sectors such as aviation [79], batteries [80], clean hydrogen technologies [81], edge and cloud technologies [82], plastics [5], processors and semiconductor technologies [83], raw materials [84], renewable and low carbon fuels [85], and solar photovoltaic (PV) [86].

In the plastics sector, the CPA was established in 2018 for the purpose of identifying and addressing the technological, operational, and economic challenges associated with expanding the European market for recycled plastics [5]. While industrial alliances such as the CPA possess a degree of autonomy to explore the feasibility of different options, it is important to emphasise that the net positive drive provided by the EU institutions frames the objectives of industrial alliances and shapes the environment within which those alliances operate [6]. For example, the CPA is presently orientated towards progressing the realisation of an objective under the Commission’s *European Strategy for Plastics* that aims to expand the European recycled plastics market to 10 million tonnes (Mt) by the year 2025 [16]. The CPA’s progress in furthering this objective will be considered below in relation to how the Alliance is contributing towards normative development for a circular plastic economy.

Finally, it should be noted that the force of the EU’s net positive drive can be felt beyond Europe through the EU’s influence on the global marketplace and the development of global rules and standards. For example, the Commission has articulated its aspiration that the EU should position itself as a global leader on plastic recycling through the promotion of global standards that safeguard the quality of recycled plastics [16, 20, 22, 25, 78]. The Commission explains that the EU has the ability to pursue green deal diplomacy firstly, by setting a “credible example”, and secondly, by employing “its influence, expertise and financial resources to mobilise its neighbours and partners to join it on a sustainable path.” [21] Sects. 1 and 3, 25] Harnessing the external influence of the EU is the central theme of the EU’s *Global Strategy for Foreign and Security Policy* [25] which reasons that interdependent problems such as environmental degradation and resource scarcity cannot easily be contained within jurisdictional borders, and therefore, such problems necessitate greater engagement with others beyond Europe [25]. Indeed, the European External Action Service (EEAS) warns that without global norms, EU interests and security are at risk [25]. As a result, the EU seeks to “participate fully in the global marketplace and co-shape the rules that govern it.” [25] p 17] The ability of the EU to effectively engage in green diplomacy around the world will depend in part upon how effectively the EU lives up to its own ideals of a circular economy within Europe [25]. For instance, the progress of the EU towards overcoming obstacles to plastic recycling could guide others and support the implementation of the *Global Plastics Treaty* that is currently being negotiated [87]. Draft provisions of the proposed treaty provide for the imposition of ecodesign requirements that would regulate the plastic content of products [88]. If a *Global Plastics Treaty* is adopted, and such provisions become international law, then the work of CPA in progressing normative development towards the facilitation of plastic recycling could contribute to the implementation of such a treaty.

If the EU is to realise its own ambitions of a European circular plastics economy then such aspirations must be operationalised through a rechannelling of conduct and expectations across European society. The task of initiating this shift in conduct and expectations has been given to the CPA which is purposed towards identifying and addressing any obstacles or barriers to the expansion of the European recycled plastics market.

## Rechannelling of Conduct by the Circular Plastics Alliance

Rechannelling of conduct is concerned with selecting the future lines along which behaviour is to be channelled [1]. In situations where a net positive drive propels a society towards new ideals and objectives, then a rechannelling of conduct is likely to be necessary in order to realign individual behaviour with newly established social expectations. The net positive drive of the EU’s circular economy agenda will require significant changes for European industry whereby certain activities are redirected from linear patterns of production, consumption, and disposal, towards more circular practices that minimise plastic waste through the retention of plastic materials in the economy for longer. The CPA has been tasked with rechannelling conduct in the plastics sector in the short term by administering a pledging campaign that secures voluntary commitments from manufacturers, brands, retailers, and recyclers in relation to reorientating their practices and operations towards circularity [16, 89]. In the longer term, the CPA is tasked with progressing the development of standards and guidelines that would better facilitate plastic recycling by regulating: product design choices; the qualitative evaluation of plastic waste and plastic recyclates; and operational practices across various phases of the plastic life-cycle and waste management processes [5, 89]. The overarching purpose the CPA’s current mandate is to foster the development of a European recycled plastics market that has the capacity to provide 10 Mt of recycled plastics by 2025 [30].

However, the voluntary commitments submitted to the CPA will not be enough to achieve the desired expansion of the European recycled plastics market. While plastic recyclers have voluntarily committed to supplying an aggregate volume of 11 Mt of plastic recyclates by 2025, manufacturers have only committed to purchasing 6.4 Mt of such recyclates by the same year [89]. Accordingly, there is a significant gap of 4.6 Mt tonnes between the supply and demand for recycled plastic [89]. This gap is understood to be the primary obstacle to establishing a functioning European recycled plastics market [5, 30]. As a result, a key objective of the CPA, is to improve the economics of plastics recycling by seeking an alignment of both the supply and demand for recycled plastic materials [30, 89].

The CPA appears to be strategically located to address the 4.6 Mt gap between supply and demand as the Alliance is a multistakeholder platform whose membership includes key actors from across plastics value chains [5]. The Alliance currently has over 300 signatories including industrial actors, business organisations, standardisation organisations, recyclers, and public authorities [8, 30]. The CPA is governed by a Steering Committee that is representative of the signatories to the Alliance. This Steering Committee organises the work programme of the CPA through the establishment of various sectoral working groups (e.g. packaging, automotive, construction, electrical and electronic equipment, and agriculture) [5, 30]. There are a number of important sectors represented in the Alliance and it is estimated that 21 Mt of plastic waste is collected from these sectors each year [30]. Accordingly, although the CPA is a voluntary initiative it would appear to be strategically positioned to identify problems, and formulate measures to rechannel behaviour and expectations across European plastics value chains and thereby maximise the volume of plastic waste that can be successfully recycled after plastic products, components, and packaging reach their end-of-life phase.

Thus far, the CPA has identified three problem areas that must be addressed to assist manufacturers in increasing their uptake of recycled plastics and reducing their reliance on virgin plastics. Firstly, plastic recyclates need to be of adequate quality and capable of adaption to the requirements of manufacturers of plastic products, components and packaging. Secondly, the supply of plastic recyclates must be sufficiently secure so as to provide a reliable source of plastic feedstock for manufacturers. Thirdly, the market for plastic recyclates must be competitive relative to the market for virgin plastics [30].

According to the CPA, addressing these problem areas will require: the development of design-for-recycling standards and guidelines for plastics; improvements to waste collection and sorting processes; and investment in better recycling technology. The CPA asserts that together, such actions could increase the volume and the quality of plastic recyclates on the European market thereby earning the confidence of plastics manufacturers. The CPA envisages that by building confidence amongst buyers of recycled plastics (i.e. plastics manufacturers), the European recycled plastics market will be able to transition from the current “push market” that anticipates *future demand*, to a “pull market” that responds to the *present demand* of buyers of plastic recyclate feedstock [30] p 8]. In this way, it is hoped that the 4.6 Mt gap between supply and demand in the market could be closed. According to the CPA, such reform within the market will require normative development in the area of design-for-recycling standards and guidelines.

The development of design-for-recycling standards and guidelines has the potential to improve the economics of plastic recycling by firstly, increasing the supply of sorted plastic waste that reaches recycling centres, and secondly, by building confidence in the quality of plastic recyclates produced thereafter. For example, the dismantling of waste products and the separation of plastic materials prior to recycling can be a difficult labour intensive process leading to losses of plastic materials [90]. Product design standards that facilitate recycling processes at the end-of-life phase can overcome such difficulties and improve the quality of plastic waste by simplifying the segregation and the sorting of plastic waste types, thereby increasing the volume and the quality of plastic waste that is forwarded to recycling facilities. Moreover, industry actors have themselves argued that the introduction of standards and guidelines that regulate the quality and the reliability of recycled plastics are enabling conditions to stimulate the expansion of the European recycled plastics market [30].

In response to the need for a normative framework that is facilitative of plastic recycling, the CPA published its *Design-for-Recycling Work Plan* in September 2021 [32]. This work plan is geared towards developing design-for-recycling standards and guidelines that: increase the volume of plastic waste that reaches European sorting facilities to 16.7 Mt by 2025; increase the volume of sorted plastic waste that reaches European recycling facilities to 11.8 Mt by 2025 [30, 32]; and improve the rate of recyclability of sorted plastic waste from 78 to 80% by 2025 [32]. It is hoped that such design-for-recycling standards and guidelines could contribute to the establishment of a stable supply of sorted plastic waste for mechanical recycling processes [32], which in turn may create favourable conditions for investment in complementary recycling methods such as chemical recycling [58].

Thus far, the CPA has identified the following areas where it considers that standardisation and guidelines are required if the obstacles to expanding the European market for plastic recyclates are to be addressed;

1. Design-for-recycling criteria with regard to future recyclability.
2. Evaluation of the quality of sorted plastic waste.
3. Evaluation of the quality of plastic recyclates.
4. Integration of plastic recyclates into products [30].

Additionally, following requests from industry seeking clarification in relation to the meaning of term ‘waste’, the CPA also published a guidance document of waste definitions that is addressed to priority sectors which have the most potential to contribute to a European recycled plastics market (e.g. agriculture; automotive; packaging; electrical and electronic equipment; and construction) [31]. It should be noted this guidance document is not a legal instrument and as a result the guidance provided therein should not be construed as authoritative from a legal perspective. Instead, the purpose of the guidance document is to provide clarity to a wide range of operators in relation to their reporting under the CPA’s monitoring system for tracking plastic recycling outputs that contribute towards the CPA’s overarching aim of expanding the European recycled plastics market to 10 Mt by 2025 [91].

The normative influence of the CPA’s work thus far, can be seen in a standardisation request that was submitted by the Commission to the European standardisation organisations, CEN [92] and CENELEC [93] in 2022, that called for the development of voluntary harmonised standards and deliverables in relation to plastic recycling and plastic recyclates [25, 94]. This standardisation request was informed by the CPA’s work in identifying obstacles and barriers to the expansion of the European recycled plastics market, and the CPA’s articulation of definitional guidance in respect of plastic waste [25, 28]. The progression of normative development towards European standardisation is an important step as harmonised standards can smooth the implementation of EU legislation, and also facilitate the free movement of goods thereby buoying the competitiveness of European operators both in Europe and globally [95].

The EU and the European Free Trade Area (EFTA) have entrusted the development of voluntary standards to the following European standardisation organisations: European Committee for Standardization (CEN), European Committee for Electrotechnical Standardization (CENELEC), and European Telecommunications Standards Institute (ESTI) [95]. These organisations have been tasked with *inter alia* developing European standards, harmonised standards, and European standardisation deliverables [95]. European standards can be defined as voluntary technical specifications that are intended for repeated or continuous application in relation to the regulation of product characteristics, production methods and processes, and the characteristics of services [95, 96]. Harmonised standards are the subset of European standards that are adopted by CEN, CENELEC, or ETSI following the submission of an initial standardisation request from the Commission [95]. Based on this distinction, the Commission’s 2022 standardisation request in respect of plastic recycling and plastic recyclates is calling on CEN and CENELEC to develop harmonised standards that would regulate aspects of product design, the quality of sorted plastic waste, and the quality of plastic recyclates [25, 28]. The standardisation request specifically calls for the development of three new harmonised standards relating to: the processes and criteria to be used for the evaluation of plastic packaging recyclability; definitions and principles to regulate design-for-recycling of plastic packaging; and the characterisation of Acrylonitrite butadiene styrene (ABS) recyclates. The standardisation request also calls for the revision of existing standards relating to: agricultural and horticultural plastic films; the characterisation of plastic wastes; and the characterisation of the following recyclates - polystyrene (PS), polyethylene (PE), polypropylene (PP), polyvinyl chloride (PVC), polyethylene terephthalate (PET) [25]. The development and revision of harmonised standards across the foregoing areas is intended to facilitate the free movement of plastics materials and recyclates in Europe, ensure high levels of interoperability, contribute to the growth of the European recycled plastics market to at least 10 Mt by 2025, while also advancing the policy goal of environmental protection by improving the management of plastic waste [25].

Harmonised standards play an important role in the EU legal order by providing operators with appropriate norms by which they may comply with the essential requirements set out in EU legislation [95]. Many EU legislative acts, including the current *Ecodesign Directive* [35] and the new proposed *Ecodesign Regulation* [36], expressly state that an operator’s compliance with *relevant* harmonised standards generates a presumption of conformity with the essential requirements under such EU legislative acts [35, 36, 95]. Accordingly, the interaction between EU legislation and harmonised standards has the dual effect of supporting the EU legal order while at the same time strengthening the normative force of harmonised standards through presumptions of conformity. This interaction between harmonised standards and EU legislation will be discussed further in the next section.

While the Commission’s 2022 standardisation request is evidence of normative progress towards a European circular plastics economy, it should be emphasised that the establishment of legal and regulatory order for a circular plastics economy is still at a relatively early stage. Currently, the adoption of harmonised standards is not feasible in all contexts as best practices for circularity are not sufficiently evolved to enable the articulation and implementation of standards. As a result, the Commission’s 2022 standardisation request has also called for the development of European standardisation deliverables [25], which are technical specifications that are intended to provide guidance to operators and possibly lay the foundations upon which future European standards can be built [95, 97]. The Commission’s standardisation request calls for the adoption of deliverables pertaining to: quality assessment of plastic recyclates; grades for the qualitative assessment of sorted plastic waste; and design-for-recycling guidelines in relation to specific types and aspects of plastic packaging, construction products, electronic and electrical equipment, road vehicles, and agricultural products [25].

It should be emphasised that because European standardisation deliverables are not harmonised standards, compliance with such deliverables would not carry a presumption of conformity with EU legislation (see below). However, that does not mean that European standardisation deliverables can be easily ignored. Such deliverables may prove to be precursors to future European standards. In this way, European standardisation deliverables may be thought of as exploring and shaping possible pathways for normative development. Indeed, a European standardisation organisation may choose to explore more than one normative pathway in relation to a particular issues through the adoption of competing technical specifications which allow for the subsequent evaluation and comparison of different solutions [97]. Therefore, European standardisation deliverables may be described as incipient standards or “pre-standards” that test possible pathways of normative development [97] p 124].

To recapitulate, the CPA has exercised an influential role in framing problems, theorising solutions, and shaping the pathway of legal and normative development towards a circular plastics economy. The CPA has responded to the Commission’s objective of expanding the European recycled plastics market to 10 Mt by 2025, by firstly administering a pledging campaign, and secondly by addressing the need for standardisation and definitional guidance in respect of plastics. This work represents an important early step in rechannelling conduct within the European plastic sector towards the EU’s vision of a circular plastics economy. Indeed, such work has paved the way for the Commission’s standardisation request that was submitted to CEN and CENELEC calling for the development of harmonised standards to facilitate plastic recyclability. While it might be argued that compliance with any future harmonised standards adopted by CEN and CENELEC would only be voluntary in nature, such standards play an important role in supporting the EU legal order and this can in turn influence the normative strength of harmonised standards in practice. Therefore, to more fully understand the significance of normative progress to date, it will be necessary to also examine how the EU’s evolving legal framework for ecodesign might interact with any future harmonised standards for plastic recyclability.

## Interaction between Harmonised Standards and the EU’s Evolving Legal Framework for Ecodesign

To understand the interaction between the *EU legal order and voluntary* harmonised standards adopted by CEN and CENELEC, it is firstly necessary to discern the distinctive quality by which law may augment the normative force of voluntary standards. According to Karl Llewellyn, what distinguishes law from other forms of social organisation is the binding nature of legal obligations. Accordingly, there is an *imperative* aspect to law, meaning that an individual can choose whether to follow non-legal norms or advice, however, an individual *must* follow the law or face the consequences [1]. In other words, the law has teeth meaning that when law comes under attack it will bite back and prevail over other interests [1]. In this way law is able to channel the conduct and uphold the expectations of different actors. To consider how conduct and expectations may be channelled to support a circular plastics economy, this article will now examine the European Commission’s proposal for a new *Ecodesign Regulation* [36], which would if adopted, replace the current *Ecodesign Directive* [35]. While the current Directive only applies to energy-related products, the proposed Regulation would extend the application of an ecodesign approach to address circularity for plastics, and indeed all physical goods (i.e. products, components, intermediate products) that are placed on the European market or put into service. However, it should be noted that there are some exceptions meaning that the scope of the proposed Regulation does not extend to: food, feed, medicinal products, living plants, animals and microorganisms, products of plants or animals relating to their reproduction, and products of human origin [36].

The overarching objective of the proposed *Ecodesign Regulation* is to establish a legal framework that sets out essential requirements for improving the environmental sustainability of products in a way that also ensures the functioning of the internal market [36]. The headline initiatives of the proposed *Ecodesign Regulation* include the establishment of: an ecodesign framework for products that are placed on the European market or put into service; a digital product passport; mandatory criteria for green public procurement; and a framework to regulate the destruction of unsold consumer products [36]. While all these complementary initiatives may come to play important roles in regulating the design of environmentally sustainable products and services, it is perhaps the ecodesign framework for products that would most obviously add some teeth to any future voluntary European standards that provide for design-for-recycling. Accordingly, for present purposes, the focus of the remainder of this article will be directed towards ascertaining how the ecodesign framework under the proposed Regulation might interact with future European standards adopted by CEN or CENELEC.

The ecodesign framework under the proposed Regulation provides that the Commission shall improve the environmental sustainability of products through the regulation of the following product aspects: durability, reliability, reusability, upgradability, reparability, maintenance, refurbishment, substances of concern, energy, resource efficiency, recycled content, remanufacturing, recycling, carbon and environmental footprint, and the expected generation of waste materials [36]. While the proposed Regulation does not explain what such requirements would specifically entail in particular contexts, the proposed Regulation does provide that more detailed and specific requirements will be elaborated through the adoption of delegated acts by the Commission [36]. In the context of the EU, delegated acts are quasi-legislative instruments that tend to be used where there is a need for frequent or recurring legislative amendments that update legal rules, standards, and best practices in light of scientific and technological progress as would likely be the case in the plastics sector where innovations are continually pursued to improve functionality, resource efficiency, and environmental performance. For clarification purposes, it should be noted that delegated acts are classified as “non-legislative” under Article 290 of the *Treaty on the Functioning of the European Union* (*TFEU*) [98], and therefore, delegated acts are not *formally* considered to be a type of EU legislation (i.e. EU legislation only denotes Regulations, Directives, or Decisions that have been adopted in accordance with the ordinary or special legislative procedures set out in Articles 289 and 294 of the *TFEU*) [98]. However, despite the formal classification of EU delegated acts as non-legislative, in practice, such delegated acts tend to exhibit many of the characteristics of secondary legislative instruments which are a significant part of the corpus of national legislation in many countries. Secondary legislation can be described as a type of legislative instrument of general application that typically establishes legally binding technical rules that give effect to more broadly articulated essential legal requirements that have been enacted by a national parliament under a statute (i.e. a ‘primary legislative’ act). Analogously, Article 290 of the *TFEU* provides that essential legal requirements must be set out in EU legislative acts (e.g. Regulations, Directives, Decisions), and that delegated acts “of general application” may be adopted to *amend* or *supplement* “non-essential” aspects of EU legislation [98 Article 290]. The quasi-legislative nature of delegated acts can also be discerned by contrasting delegated acts with EU implementing acts that are executive in nature. Under the *TFEU*, while both delegated acts and implementing acts are of general application and legally binding, implementing acts can be distinguished on the basis that they do not amend or supplement EU legislative acts. Instead, implementing acts are instruments that merely give effect to EU legislation by establishing uniform conditions for the implementation of EU law [99]. Accordingly, despite their formal non-legislative status, delegated acts tend to be understood as a type of secondary legislation, because in practice, they are often utilised as a flexible law-making instrument by which to elaborate non-essential aspects of EU legislation without having to fulfil all the requirements of legislative procedure [100, 101].

Under the proposed *Ecodesign Regulation*, delegated acts have been designated as the legal medium through which the Commission will articulate many of the specific legal obligations that would be mandated by that Regulation. Significantly, the proposed Regulation would empower the Commission to adopt delegated acts that are capable of creating legal obligations requiring that categories of products placed on the European market or put into service must conform to certain ecodesign and product performance standards [36]. The creation and imposition of such obligations could extend along entire plastic value chains to regulate the decisions of manufacturers, authorised representatives of manufacturers, importers, distributers, dealers, economic operators that place products on the market (e.g. in relation to labelling and the provision of information, monitoring and reporting), fulfilment service providers, and online marketplaces, and search engines [36]. Accordingly, despite their formal non-legislative status, in practice such delegated acts could create and impose *legal* obligations on a wide range of actors [101].

The next matter to consider is how delegated acts adopted pursuant to the proposed Regulation might interact with European standards that could be adopted by CEN or CENELEC in the future. Such interactions can be analysed firstly in relation to those situations where a delegated act amends the proposed Regulation, and secondly those situations where a delegated act supplements the proposed Regulation.

The Commission’s powers of amending the proposed Regulation by delegated acts are confined to two situations where it may be necessary to substitute or add to international standards that have been stipulated in, and thereby incorporated into, the proposed Regulation. For example, under Article 9(1)(c) of the proposed Regulation, the Commission is empowered to replace or add to the stipulated international standard (i.e. ISO/IEC 15459-2:2015) by incorporating other European or international standard(s) that relate to data carriers and unique identifiers that are to be contained in product passports [36]. The Commission is similarly empowered to amend Article 11(1) of the proposed Regulation to replace or add to the stipulated international standard (i.e. ISO/IEC 15456-2:2015) that would apply to unique operator identifiers and unique facility identifiers [36]. Accordingly, the Commission’s powers to amend the proposed Regulation are confined to just two situations where the stipulated international standard may be replaced or added to in light of technical and scientific progress. However, neither of these two situations are directly concerned with matters pertaining to the Commission’s 2022 standardisation request regarding plastic recycling and recyclates [25, 28]. As a result, it is the Commission’s ability to supplement the proposed Regulation that appears most likely to give rise to future interactions with European standards for plastic recycling and recyclates.

The Commission has been vested with a much greater array of powers to adopt delegated acts that supplement the proposed Regulation [36]. It is important to emphasise that such powers are supplementary, meaning that they may only be exercised to augment specified provisions under the proposed Regulation. The matters over which the Commission can exercise such delegated powers are mandated by the proposed Regulation, which also prescribes the content and form that delegated acts must take [36]. The matters over which the Commission may adopt delegated acts include: the determination of ecodesign requirements for particular product groups; product performance requirements; information requirements; labelling; digital product passports; methods of verification (i.e. tests, measurements, and calculations); conformity assessment procedures; and the establishment of prohibitions in relation to the destruction of certain types of unsold products [36]. The use of delegated powers in this way provides a flexible means of elaborating technical requirements across what is potentially a wide array of product groups and contexts. Indeed, the important role that delegated acts play within the EU legal order was recognised in the EU *Institutions Interinstitutional Agreement on Better Law-Making* which acknowledged the contribution that such instruments make to ensuring that the EU legislative process is efficient and that legislation is kept up to date [102].

If the Commission were to adopt delegated acts to supplement the proposed *Ecodesign Regulation* then such delegated acts could interact with European standards relating to matters such as: product design; the quality of sorted plastic waste; and the quality of plastic recyclates [25]. Under the proposed Regulation such interactions would be likely to occur firstly, during the formulation of delegated acts by the Commission, and secondly, through the operation of presumptions of conformity [36]. In relation to the formulation of delegated acts, Article 5(4)(a) of the proposed Regulation provides that when the Commission seeks to establish ecodesign requirements through delegated acts, regard must be had to any relevant European and international standards. This is a familiar requirement across EU product legislation and the Commission has adopted a number of delegated acts in the past that expressly refer to European standards covering *inter alia*: operator requirements pertaining to technical documentation; labelling information; and compliance and verification processes [33, 34]. Accordingly, if CEN or CENELEC were to adopt harmonised standards in relation to plastic recycling and recyclates, then under the proposed *Ecodesign Regulation* the Commission would be obligated to have regard to such standards when formulating delegated acts pursuant to Article 5 of the proposed Regulation.

The second point of interaction between supplementary delegated acts and harmonised standards concerns the matter of conformity to EU legislative requirements. While harmonised standards are voluntary in character, compliance with such standards can carry a presumption of conformity with *relevant* EU legislation [103]. The origin of such presumptions of conformity between harmonised standards and EU legislation can be traced back to circumstances created by the *Cassis de Dijon* case where the Court of Justice of the European Union (CJEU) recognised the principle of mutual recognition [104]. This principle provides that in the absence of harmonised EU legislation, goods that are lawfully marketed in one Member State must be allowed to enter other Member States without restriction unless the imposition of national restrictions can be justified under certain mandatory or essential policy requirements (e.g. public policy, public security, public health, consumer protection) [104]. While the decision in *Cassis* helped to facilitate the free movement of goods, it did not address the matter of how Member States were supposed to trust products that had been manufactured in other jurisdictions [24]. The issues created by *Cassis* led to a “New Approach” to EU legislation in the 1980s which saw a greater emphasis on addressing essential policy matters through EU legislation while leaving the development of harmonised technical specifications to the European standardisation organisations (i.e. CEN, CENELEC, ETSI) [24 p 7, 103 preamble] Many of the EU legislative acts adopted since that time expressly provide that an operator’s compliance with harmonised standards carries a presumption of conformity with relevant requirements under EU legislation. Such presumptions of conformity assist operators in accessing markets across Member States, while also ensuring that Member States have confidence in the quality of the products entering their jurisdictions [103]. In this way, the interaction of EU legislation and harmonised standards was intended to guarantee compliance with essential requirements provided for under EU legislation [103]. It follows that if harmonised standards are adopted on foot of the Commission’s 2022 standardisation request in relation to plastic recycling and recyclates [25, 28], then such standards could come to play an important role in regulating compliance with the essential sustainability requirements of the proposed Ecodesign Regulation and any delegated acts adopted thereto. In other words, if the Commission were to adopt a future delegated act pertaining to the ecodesign of plastic products, then relevant harmonised standards developed by CEN or CENELEC could come to demonstrate compliance with the proposed Regulation and ultimately determine a product’s access to the European market.

Accordingly, if the European Parliament and the Council choose to adopt the proposed *Ecodesign Regulation* then that instrument may provide the legal teeth which Karl Llewellyn considered were necessary to channel conduct and expectations, and avoid or minimise conflict and disputes within society [1]. While voluntary commitments, declarations, and pledges made by corporate actors are often not fulfilled [105], the proposed *Ecodesign Regulation* and any delegated acts adopted thereto, could create legal obligations in respect of a wide range of actors across plastic value chains, and such obligations would have a legal bite that could be enforced in the event of non-compliance [36]. However, it should also be noted that while a capacity for legal enforcement is usually necessary to uphold the expectations of society, the aftermath of the *Cassis de Dijon* case [104] shows that the law is not always able to perform *every* function necessary to avoid trouble and conflict [24]. The decision in *Cassis* did not address the issue of Member States lacking trust in some of the products entering their jurisdictions. Instead that issue was eventually addressed through voluntary harmonised standards which carry a presumption of conformity with relevant requirements of EU legislation [24].

It remains to be seen if the proposed *Ecodesign Regulation* is adopted and in what form [36], and what related delegated acts and European standards might emerge in the future. However, it is clear that normative development concerning plastic recycling and recyclates is progressing. In this regard, the work of the CPA has played an important part, firstly, by identifying obstacles to the expansion of the European recycled plastics market [30], and secondly, by mapping the areas in need of standardisation if such obstacles are to be overcome [32]. As explained above, such work helped to inform the standardisation request submitted by the Commission to CEN and CENELEC in 2022. If harmonised standards are adopted on foot of this request, then they could be applied to demonstrate conformity with the proposed *Ecodesign Regulation*, and could also influence the formulation of any delegated acts adopted thereto. Accordingly, the CPA is playing an important role in the rechannelling of conduct and expectations towards a European circular economy for plastics.

There is one final point to be made about the output of the CPA that could be relevant in the event that the proposed *Ecodesign Regulation* is adopted. Article 16 of the proposed Regulation would impose a duty on the Commission to prioritise the regulation of product groups that have the most potential to contribute to EU objectives such as the establishment of a European circular plastics economy [36]. In this regard, it should be noted that the CPA has already identified 26 priority product categories across a number of sectors (e.g. agriculture, construction, electronic and electrical equipment, and packaging) which the Alliance considers could benefit from design-for-recycling [32]. These product categories are cumulatively estimated to account for about 18.4 Mt of the plastic waste that is available for collection in Europe each year [32]. The prioritisation of these product categories by the CPA was based on criteria pertaining to the ability of such categories to contribute to the Commission’s target of expanding the European recycled plastics market to 10 Mt by the year 2025. The criteria included factors such as: the quantities of plastics (in tonnes) placed on the EU market each year; the quantities of plastic waste collected and sorted each year; whether design-for-recycling standards or guidelines could be developed; and whether design-for-recycling standards or guidelines already exist but could be improved [32]. Accordingly, if the proposed *Ecodesign Regulation* is adopted then the sectoral knowledge and experience accrued by CPA could assist the Commission in prioritising the most suitable product categories for the application of ecodesign requirements.

## Conclusion

While certain arenas of human life such as technological development may on the surface appear to be entirely technical in nature, by delving deeper it can be seen that law tends to permeate human culture to perform important functions that contribute to the minimisation of conflicts and disputes within societies [1]. The global pollution and waste crisis presents complex environmental and economic challenges within which there is the potential for conflicts and disputes to emerge and proliferate amongst a broad range of societal actors and interests. Accordingly, it is perhaps not surprising that the evolving EU legal and normative framework for a circular plastics economy has manifested as a polycentric governance arrangement that includes the EU institutions, the CPA, and European standardisation organisations (i.e. CEN and CENELEC). By applying the theoretical framework of Karl Llewellyn’s law-jobs theory to this governance context, it is possible to analyse how each of the foregoing governance bodies perform certain legal functions (i.e. law-jobs) that contribute to the development of regulatory order for a European circular plastics economy. This article has shown that the EU institutions, in particular the Commission, provide a policy framework and impetus (e.g. net positive drive) through which normative development for a European circular plastics economy takes place. While there are a plurality of conceptions and articulations in relation to what a circular plastics economy should be [13–15, 75], it is the EU vision as set out in its circular economy agenda [16, 19–21] that is currently setting the parameters for normative development by the CPA and the European Standardisation organisations (e.g. CEN and CENELEC). Within the EU framework, the CPA and the European standardisation organisations pursue normative development by identifying regulatory and economic challenges and developing norms that respond to such challenges. Where a number of governance bodies cooperate and contribute to the provision of order in this way, then it may be said that such bodies are part of a polycentric governance arrangement [10]. In this case, the CPA, European standardisation organisations and the EU institutions “generate some degree of ‘polycentric order’ in their patterns of behaviour, interactions, and outcomes” [106] p 29]. According to Stephan, Marshall, and McGinnis a statement that a polycentric governance arrangement *provides governance* can be justified where the actions of multiple decision-centres influence the actions or choices of each other [106]. In the case of a European circular economy for plastics, this article has shown that there is clear evidence of cooperation, coordination, and influence between the EU institutions, European standardisation organisations, and the CPA. Most notably, so far, the work of the CPA has led to the Commission submitting a standardisation request to the European standardisation organisations calling for the development of harmonised standards and European standardisation deliverables in relation to plastic recycling and recyclates. Any harmonised standards adopted by CEN or CENELEC on foot of this request, may in time interact with the proposed *Ecodesign Regulation* during the formulation of delegated acts by the Commission, or by the application of presumptions of conformity with the proposed Regulation. Accordingly, the CPA, European standardisation organisations, and the EU institutions each perform specific functional roles that contribute to the provision of order for a European circular plastics economy, while still maintaining internal decision-making autonomy within their own particular domains [107]. A theoretical implication of this governance configuration is the inadequacy of traditional legal paradigms that only focus on the formal law-making processes that flow through the European Parliament, Council, and Commission. Instead, governance for a European circular plastics economy presents legal scholars with a more complex and polycentric governance context that additionally includes the CPA and European standardisation bodies. Karl Llewellyn’s law-jobs theory provides a heuristic legal perspective from which it is possible to capture and analyse the functional roles of the EU institutions, European standardisation bodies, and the CPA [11]. This is an important mode of analysis given the influence of the CPA in firstly, identifying obstacles (e.g. technological, operational, economic) to the achievement of European policy objectives, and secondly, progressing the development of product design standards and guidelines.

In addition to supporting the implementation of the proposed *Ecodesign Regulation*, the adoption of harmonised standards for plastic recycling and plastic recyclates, could also have practical implications for the operation of other EU legislation such as the Waste Electrical and Electronic Equipment (WEEE) Directive [108], and the End-of-Life Vehicles (ELV) Directive [109]. For instance, if harmonised standards are adopted by CEN and CENELEC pursuant to the Commission’s 2022 standardisation request [25], then such ecodesign standards could help to facilitate the fulfilment of legal obligations pertaining to the separate collection and proper treatment of electrical and electronic waste under Articles 5 and 8 and Annex VII of the WEEE Directive [108]. Similarly, better ecodesign design practices could also assist the fulfilment of legal obligations requiring the removal and recycling of plastic components from vehicles (e.g. bumpers, containers, seating, dashboards) under the End of Life Vehicles Directive [109]. It should also be noted that the normative work initiated by the CPA, and the evolving EU regulatory order for a circular plastics economy, could also have knock-on effects beyond Europe given the influence of EU norms in the global marketplace. Indeed, the development of pioneering norms in key jurisdictions such as the EU often have significance beyond their borders through normative diffusion. There are numerous examples of successful environmental norms being transplanted around the world (e.g. the precautionary principle originating from Germany, and environmental impact assessment from the United States) [110–112]. Accordingly, if the EU can realise its own sustainability ambitions by harnessing the abilities of a broad coalition of actors across plastic value chains, then Europe could be in a position to become a global leader in establishing a circular plastics economy.

The example of the CPA shows the importance of mobilising key value chain actors towards the achievement of circular ambitions. As a result, the CPA may provide lessons for other industrial alliances such as the Alliance for Zero-Emission Aviation (AZEA) which is currently undertaking analysis to identify the need for standards to support the certification of hydrogen and electric aircraft [79, 113]. However, it should be noted that while this article has shown that the CPA is playing an important role in the initiation of normative development within the evolving EU regulatory order for a circular plastics economy, the role of the CPA is not uncontroversial and as a result further study is recommended. There has been criticism of the exclusive role accorded to the CPA in the early stages of the standardisation process. For example, in recent years, coalitions of non-governmental organisations (Environmental Coalition on Standards, European Environmental Bureau, Zero Waste Europe, and Rethink Plastics Alliance) [114–117] have argued that contrary to the Vademecum on European Standardisation [26], the Commission gave the CPA a privileged role in the drafting process for its 2022 standardisation request [27]. The prioritisation of the CPA, which in turn leads to the marginalisation of other viewpoints, runs the risk of limiting stakeholder consultations to just a narrow range of technocratic inputs. Such a situation is not reflective of the plurality of conceptions, strategies, and aspirations that contribute to evolving discourses on the circular plastics economy. Furthermore, given the important role that standardisation plays in supporting the implementation of EU legislation, the formulation of a standardisation request and the objectives contained therein, cannot be said to be an entirely depoliticised process [13]. Accordingly, the important functional role of the CPA within the EU regulatory order for a circular plastics economy indicates that more study is needed in relation to the input and throughput legitimacy of the CPA and other industrial alliances within the European standardisation process [118, 119]. Further study is also needed to assess the output of the Commission’s 2022 standardisation request as that process is still ongoing through CEN and CENELEC [27]. Lastly, it is suggested that future research might undertake a comparative analysis of the CPA and other industrial alliances within the European standardisation process and the wider EU regulatory order. Such analyses could deepen our understanding of the normative role that industrial alliances are currently playing within the EU.

## Data Availability

Not applicable.

## Abbreviations

ABS: Acrylonitrite butadiene styrene
CEN: European Committee for Standardization
CENELEC: European Committee for Electrotechnical Standardization
CJEU: Court of Justice of the European Union
CPA: Circular Plastics Alliance
EFTA: European Free Trade Area
ETSI: European Telecommunications Standards Institute
EU: European Union
GDP: Gross Domestic Product
Mt: Million tonnes
PE: Polyethylene
PET: Polyethylene terephthalate
PP: Polypropylene
PS: Polystyrene
PV: Photovoltaic
PVC: Polyvinyl chloride
TFEU: Treaty on the Functioning of the European Union
UNCTAD: United Nations Conference on Trade and Development
